# An artificial intelligence network‐guided signature for predicting outcome and immunotherapy response in lung adenocarcinoma patients based on 26 machine learning algorithms

**DOI:** 10.1111/cpr.13409

**Published:** 2023-02-23

**Authors:** Nan Zhang, Hao Zhang, Zaoqu Liu, Ziyu Dai, Wantao Wu, Ran Zhou, Shuyu Li, Zeyu Wang, Xisong Liang, Jie Wen, Xun Zhang, Bo Zhang, Sirui Ouyang, Jian Zhang, Peng Luo, Xizhe Li, Quan Cheng

**Affiliations:** ^1^ Department of Neurosurgery, Xiangya Hospital Central South University Changsha China; ^2^ College of Life Science and Technology Huazhong University of Science and Technology Wuhan China; ^3^ National Clinical Research Center for Geriatric Disorders, Xiangya Hospital Central South University Changsha China; ^4^ Department of Neurosurgery, The Second Affiliated Hospital Chongqing Medical University Chongqing China; ^5^ Department of Interventional Radiology The First Affiliated Hospital of Zhengzhou University Zhengzhou China; ^6^ Department of Oncology, Xiangya Hospital Central South University Changsha China; ^7^ Division of Neuroscience and Experimental Psychology, Faculty of Biology, Medicine and Health University of Manchester Manchester UK; ^8^ Department of Thyroid and Breast Surgery, Tongji Hospital Tongji Medical College of Huazhong University of Science and Technology Wuhan China; ^9^ Department of Oncology, Zhujiang Hospital Southern Medical University Guangzhou China; ^10^ Department of Thoracic Surgery, Xiangya Hospital Central South University Changsha China; ^11^ Hunan Engineering Research Center for Pulmonary Nodules Precise Diagnosis & Treatment Changsha China

## Abstract

The immune cells play an increasingly vital role in influencing the proliferation, progression, and metastasis of lung adenocarcinoma (LUAD) cells. However, the potential of immune cells' specific genes‐based model remains largely unknown. In the current study, by analysing single‐cell RNA sequencing (scRNA‐seq) data and bulk RNA sequencing data, the tumour‐infiltrating immune cell (TIIC) associated signature was developed based on a total of 26 machine learning (ML) algorithms. As a result, the TIIC signature score could predict survival outcomes of LUAD patients across five independent datasets. The TIIC signature score showed superior performance to 168 previously established signatures in LUAD. Moreover, the TIIC signature score developed by the immunofluorescence staining of the tissue array of LUAD patients showed a prognostic value. Our research revealed a solid connection between TIIC signature score and tumour immunity as well as metabolism. Additionally, it has been discovered that the TIIC signature score can forecast genomic change, chemotherapeutic drug susceptibility, and—most significantly—immunotherapeutic response. As a newly demonstrated biomarker, the TIIC signature score facilitated the selection of the LUAD population who would benefit from future clinical stratification.

## Graphical abstract

In this study, by analysing single‐cell RNA sequencing (scRNA‐seq) data and bulk RNA sequencing data, the tumour‐infiltrating immune cell (TIIC) associated signature was developed based on 26 machine learning algorithms. The performance of the TIIC signature score in predicting prognosis and immunotherapy response in LUAD patients was systematically excavated.

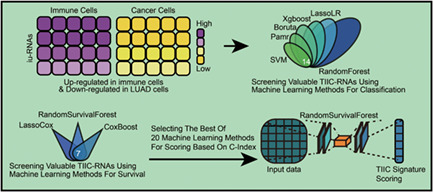

## 1 INTRODUCTION

Lung adenocarcinoma (LUAD) has a rising incidence worldwide.^1^, ^2^ In addition to conventional treatments such as surgery and chemotherapy, the accumulation of molecular knowledge through emerging technologies allows the discovery of molecular targets for LUAD and the application of targeted therapy. A large propor tion of LUAD patients possess specific genomic aberrations, including EGFR, ALK, HER2, and so on, and benefit from targeted therapies.^3^ Despite the advances in novel therapies, LUAD is still a global threat, with overall survival of fewer than 5 years.^4^ Besides, the high resistance to current therapies in LUAD patients also augments the need to explore and develop new effective treatments. With the advent of immunotherapy, a new era of cancer treatment has dawned along with unprecedented and promising therapeutic responses in a wide range of solid tumours. Immunotherapy targeting PD‐1/PD‐L1 and CTLA‐4 has validated therapeutic efficacy and has been approved for clinical application.^5^ However, the beneficiaries are limited to only a minority of LUAD patients.^6^, ^7^ It hence set off a wave of research into how to reinvigorate the immune system and respond to immunotherapy effectively.

The tumour microenvironment (TME) is the complex multi‐cellular environment, which comprises immune cells, stromal cells, extracellular matrix, secreted molecules, and the blood and lymphatic vascular networks.^8^ TME plays a crucial role in regulating tumour growth and mediating cell–cell interactions. The immune activity of the TME is the key to the effect of immunotherapy, making therapies targeting components of TME, including tumour‐associated macrophages (TAMs), dendritic cells (DCs), cancer‐associated fibroblasts (CAFs), and so on, become research spotlights in recent years.^8^ In the era of massive data, there is a wealth of information hidden beneath, waiting to be mined through various new technologies. Single‐cell transcriptome enables a comprehensive analysis of the diversified cells in TME with high resolution.^9^ As a major part of artificial intelligence, machine learning (ML) contributes to building reliable models based on big data for precise prediction.^10^ With the help of these novel technologies, we could dig deeper into the therapy resistance mechanisms from different levels, including transcriptional, translational, and epigenetic levels, and find more clues to improve immunotherapy efficacy.^11^, ^12^

In this study, by analysing single‐cell RNA sequencing (scRNA‐seq) data and bulk RNA sequencing data, the tumour‐infiltrating immune cell (TIIC) associated signature was developed based on 26 ML algorithms. The performance of the TIIC signature score in predicting prognosis and immunotherapy response in LUAD patients was systematically excavated.

## 2 MATERIALS AND METHODS

The detailed method is provided in the Supplementary material.

### 2.1 Collection and pre‐processing of the LUAD transcriptome data

Transcriptome data and clinical data of LUAD patients were accessed from The Cancer Genome Atlas (TCGA) and Gene Expression Omnibus (GEO) databases. 1404 samples from five datasets were included, including 502 patients from the TCGA LUAD dataset, 226 patients from the GSE31210 dataset, 106 patients from the GSE37745 dataset, 128 patients from the GSE50081 dataset, and 442 patients from the GSE68465 dataset.

### 2.2 Collection and pre‐processing of the LUAD scRNA‐seq data

The scRNA‐seq dataset included 43 clinical biopsies obtained from 28 lung adenocarcinoma patients and were accessed from European Nucleotide Archive (ENA, https://www.ebi.ac.uk/ena/browser/view/PRJNA591860). Copy number variants (CNVs) are widely used in cancer research to identify malignant cells. We estimated CNVs for single cells using the R package ‘infercnv’,^13^ which calculates a moving average of expression values on each chromosome for each cell and then compares this to expression values previously collected in the literature for normal reference cells to estimate CNVs for each cell. The cell clustering and dimension reduction were further performed using the R package ‘Seurat’.^14^ Subsequently, the principal component analysis (PCA), ‘FindNeighbors’, and ‘FindClusters’ functions were applied to identify the cell clusters. Epithelial/cancer markers (EpCAM+, EPCAM) and immune markers (CD45+, PTPRC) were used for the initial identification of cell types. Next, immune cells were further subdivided into subtypes using the ‘CellTypist’ function.^15^ We used the ‘FindMarkers’ function to identify significantly differentially expressed genes (DEGs) between immune cells and neoplastic cells.

### 2.3 Biological peculiarities of the TIIC signature score at the single‐cell level

The TIIC signature score was also constructed in the scRNA‐seq dataset. The Biological peculiarities were performed using gene set variation analysis (GSVA) and gene set enrichment analysis (GSEA). Cell–cell communication pattern was determined using the R package ‘CellChat’^16^ and the R package ‘iTalk’^17^ to infer, analyse and visualize the different receptor‐ligand modules between high and low TIIC score groups of malignant cells and immune cells. scMetabolism^18^ was used to quantify the activity of related metabolic pathways based on the Kyoto Encyclopedia of Genes and Genomes (KEGG) and REACTOME terms.

### 2.4 Development of tumour‐infiltrating immune cell‐related signature. ^19^, ^20^

The detailed methods of this part are provided in the supplementary file.

### 2.5 Functional annotation of the TIIC signature score.^21^, ^22^, ^23^, ^24^, ^25^, ^26^, ^27^, ^28^, ^29^, ^30^, ^31^, ^32^, ^33^

The detailed methods of this part are provided in the supplementary file.

### 2.6 Immunotherapeutic response prediction.^34^, ^35^, ^36^, ^37^, ^38^, ^39^, ^40^, ^41^, ^42^, ^43^, ^44^, ^45^

The detailed methods of this part are provided in the supplementary file.

### 2.7 Multi‐omics alteration characteristics of the TIIC signature score

Genomic alterations (recurrently amplified and deleted regions) were determined using the GISTIC 2.0 analysis (https://gatk.broadinstitute.org). The R package ‘maftools’ was used to calculate the TMB.^46^ The fraction of genome alteration (FGA), the fraction of genome gained (FGG), and the fraction of genome lost (FGL), were defined as total CNV/all bases, gain bases/all bases, and loss bases/all bases, respectively. The mutually occurred and exclusive mutations were detected using the CoMEt algorithm.

### 2.8 Drug susceptibility prediction

The largest publicly available Genomics of Drug Sensitivity in Cancer (GDSC; https://www.cancerrxgene.org) database was used to predict each sample's chemotherapeutic response. The R package oncoPredict was used for performing the prediction process by calculating the drug sensitivity which is similar to the half maximal inhibitory concentration (IC_50_).^47^ The drug response was also generated from the Cancer Therapeutics Response Portal (CTRP; https://portals.broadinstitute.org/ctrp) and Profiling Relative Inhibition Simultaneously in Mixtures (PRISM; https://depmap.org/portal/prism) databases. The area under the curve (AUC) values measured drug sensitivity with R package pRRophetic.^48^ Besides, Complement Map (CMap; http://www.complement.us/labweb/cmap) database was also used for drug prediction^49^, ^50^ with R package PharmacoGx.

### 2.9 Multiplex immunofluorescence staining in LUAD samples

We obtained the tissue microarray from the Outdo Biotech company (HLugA180Su08, Shanghai, China) and the ethics was approved. Anti‐CFL1 antibody (rabbit, 10960‐1‐AP), anti‐PABPC1 antibody (rabbit, 10970‐1‐AP), anti‐CCDC85B antibody (rabbit, 18282‐1‐AP, Proteintech, China), anti‐PFN1 (mouse, 67390‐1‐Ig, Proteintech, China), anti‐HSP90AA1 (rabbit, 13171‐1‐AP, Proteintech, China), anti‐HMGB1 (rabbit, 10829‐1‐AP, Proteintech, China) and anti‐RPS15 (rabbit, 14957‐1‐AP, Proteintech, China) were used as the primary antibody. The secondary antibody (GB23301, GB23303, Servicebio, China) was subsequently used for incubation and tyramide signal amplification (TSA) (FITC‐TSA, CY3‐TSA, 594‐TSA, 647‐TSA, Servicebio, China). The nuclei were stained with 4′,6‐diamidino2‐phenylindole dihydrochloride (DAPI). The Pannoramic Scanner (3D HISTECH, Hungary) was used for image capture. The quantification of the stained markers was performed.

## 3 RESULTS

### 3.1 Identification of the TIIC‐RNAs at the single‐cell level

**FIGURE 1:**
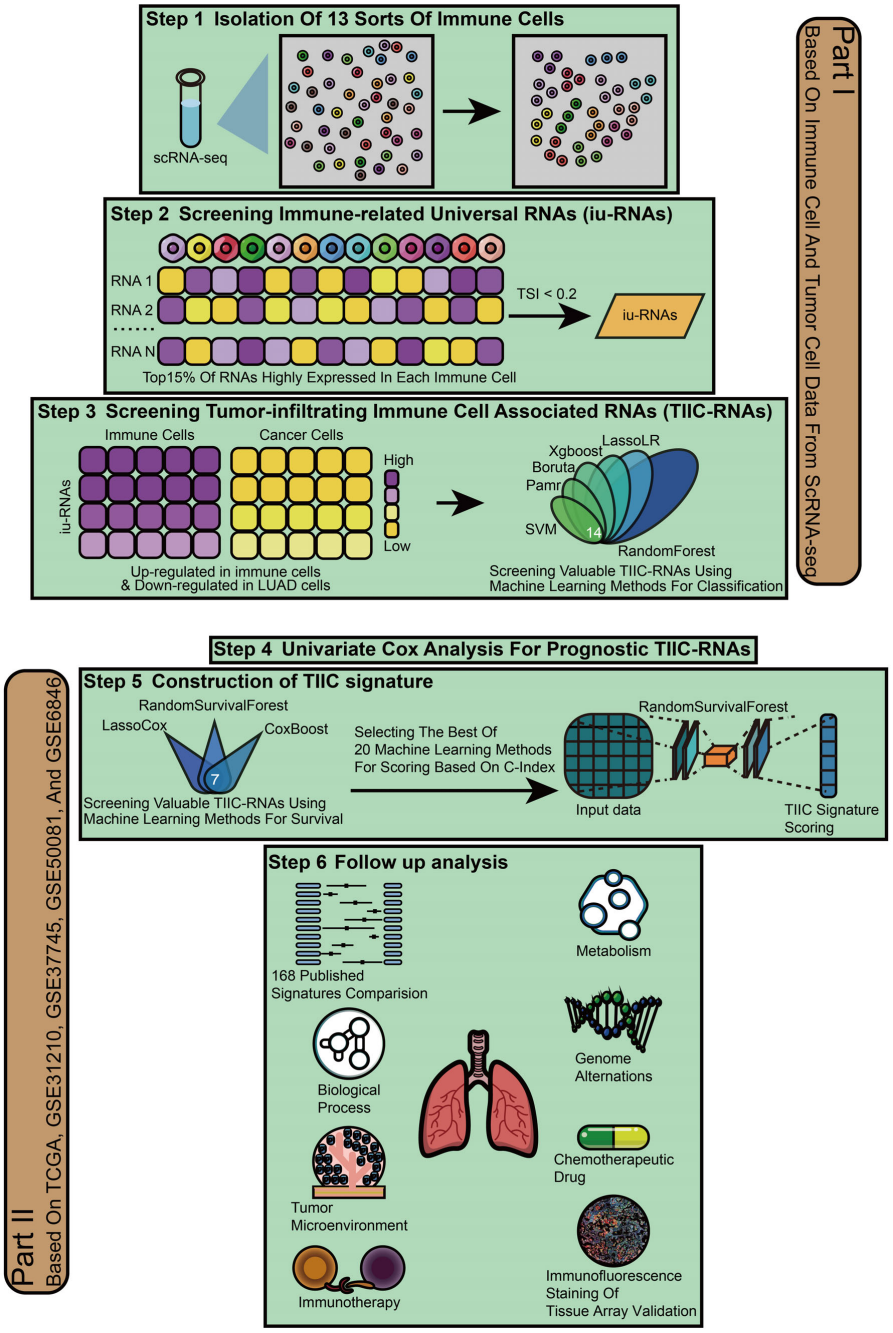
The flowchart of the overall study.

**FIGURE 2:**
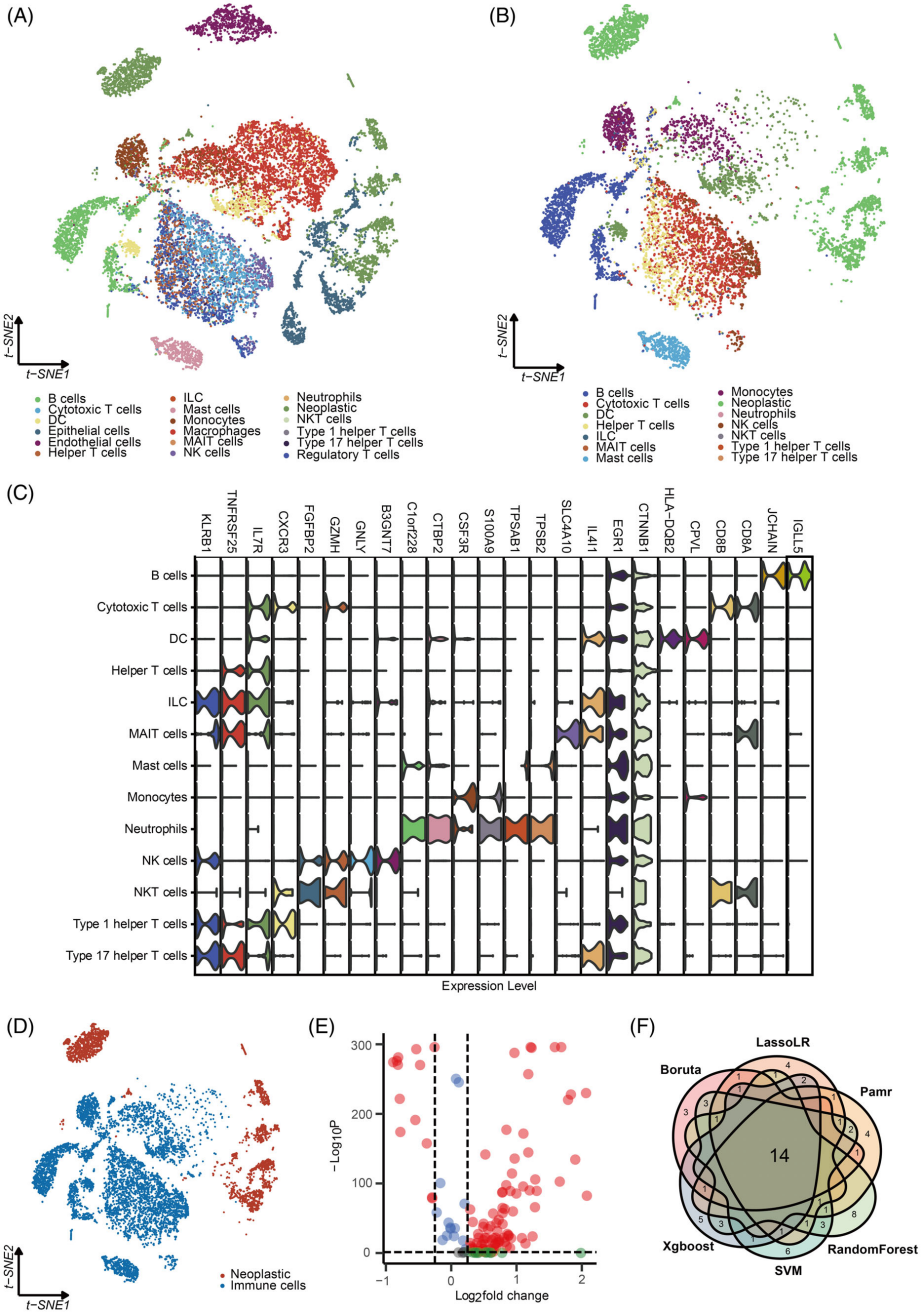
Identification of the TIIC‐RNAs at the single‐cell level. A. t‐SNE plot of the identified microenvironment cells and LUAD cells. B. t‐SNE plot of the identified LUAD cells and 13 types of immune cells. C. Vlnplot of the differentially expressed genes among the identified immune cells. D. t‐SNE plot of the identified immune cells and LUAD cells. E. Volcano plot of the differentially expressed genes between two TIIC signature groups. F. Venn plot shows the intersected genes identified by six ML algorithms for classification.

The workflow of the study is shown in Figure 1. Based on the LUAD scRNA‐seq dataset, LUAD cells and 17 types of microenvironment cells were identified (Figure 2A). LUAD cells and 13 types of immune cells were selected for further analysis (Figure 2B). The top 15% of RNAs expressed in each immune cell were identified as potential immune‐related RNAs for each immune cell type, for a total of 4048 RNAs. 135 immune‐related RNAs were further determined as iuRNAs based on the TSI score. The DEGs among the immune cells were shown in Figure 2C to prove the accuracy of the defined cells. The DEGs between immune cells and LUAD cells (Figure 2D) were shown in Figure 2E. 68 DEGs, significantly upregulated in immune cells compared to LUAD cells, were defined as TIIC‐RNAs. Six ML algorithms for classification, including Boruta, Xgboost, LassoLR, SVM, RF, and Pamr, were applied to identify the 14 most valuable TIIC‐RNAs on the basis of the previous TIIC‐RNAs.

### 3.2 Construction of the TIIC signature score

**FIGURE 3:**
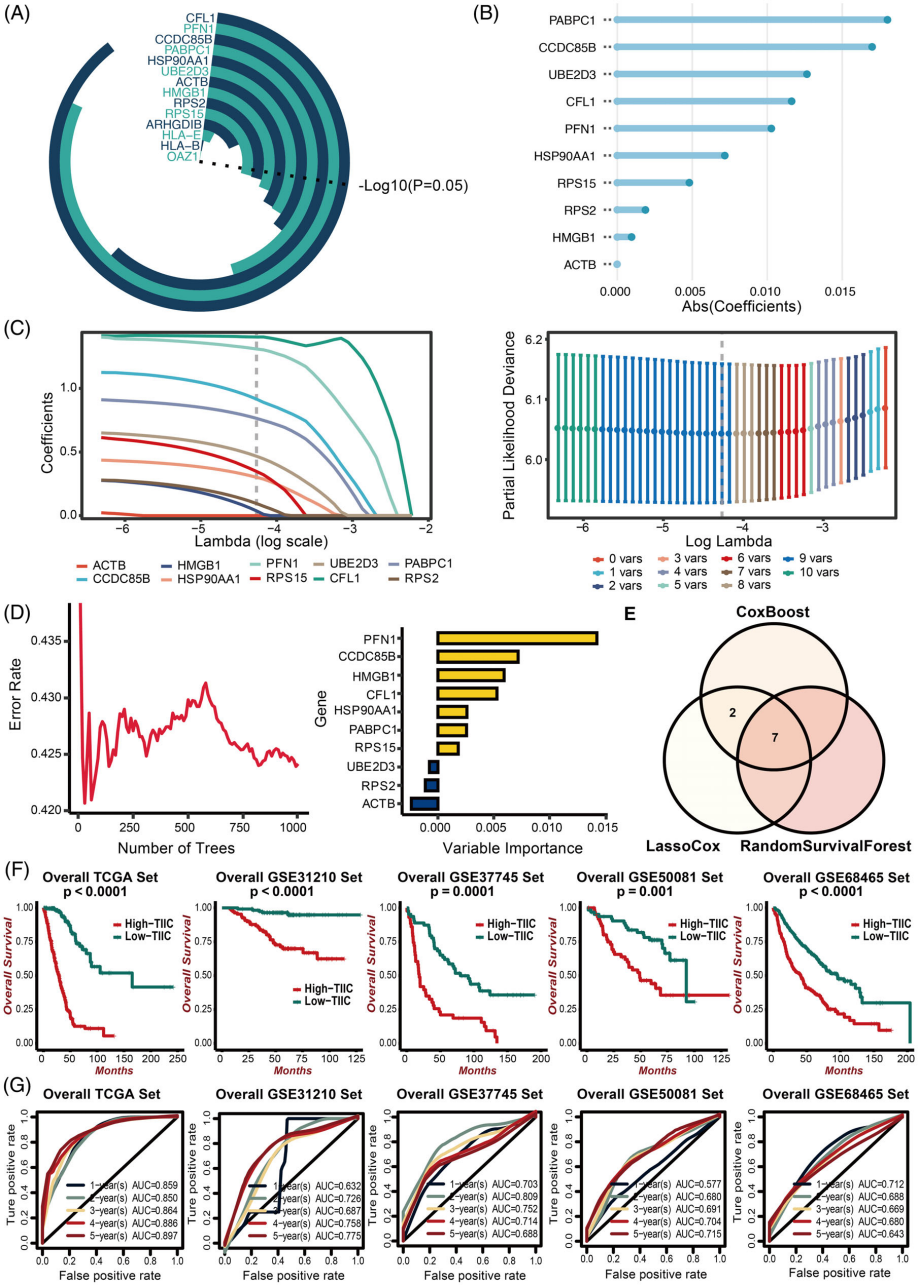
Development of the risk signature based on ML. A. Univariate Cox regression analysis of the 14 intersected genes. B. Dimension reduction of the 10 prognostic genes by the CoxBoost algorithm. C. Dimension reduction of the 10 prognostic genes by the Lasso algorithm. D. Dimension reduction of the 10 prognostic genes by Random Survival Forest algorithm. E. Venn plot shows the intersected prognostic genes identified by three ML algorithms for survival. F. Kaplan–Meier survival curves of the TIIC signature score regarding OS in the TCGA, GSE31210, GSE37745, GSE50081, and GSE68465 datasets. G. Time‐dependent ROC curves of the TIIC signature score regarding 1‐, 2‐, 3‐, 4‐, and 5‐year OS in the TCGA, GSE31210, GSE37745, GSE50081, and GSE68465 datasets.

Univariate Cox proportional hazards regression analysis was also performed to explore the prognostic value of TIIC‐RNAs regarding OS in LUAD patients. It turned out that 10 TIIC‐RNAs were identified in the TCGA dataset (Figure 3A). Three ML algorithms for survival, including LassoCox (Figure 3B), CoxBoost (Figure 3C), and RSF (Figure 3D), were further applied to determine seven intersecting prognostic TIIC‐RNAs (Figure 3E). Then 20 ML algorithms for scoring were used to determine the most reliable model based on comprehensive C‐index of external validation datasets (Figure S1). The TIIC signature score was developed based on the seven prognostic TIIC‐RNAs using the RSF algorithm with the best performance among 20 ML algorithms for scoring. LUAD patients with high TIIC signature scores presented worse survival outcomes in the TCGA, GSE31210, GSE37745, GSE50081, and GSE68465 datasets (Figure 3F). The time‐dependent ROC curves quantified by AUC values at 1, 2, 3 years, 4 years, and 5 years of OS proved the prognostic value of the TIIC signature score in the TCGA (0.859, 0.85, 0.864, 0.886, 0.897), GSE31210 (0.632, 0.726, 0.687, 0.758, 0.775), GSE37745 (0.703, 0.809, 0.752, 0.714, 0.688), GSE50081 (0.577, 0.68, 0.691, 0.704, 0.715), and GSE68465 (0.712, 0.688, 0.669, 0.68, 0.643) datasets (Figure 3G).

### 3.3 The biological peculiarities of the TIIC signature score at the single‐cell level

The TIIC signature score was also further developed in the LUAD scRNA‐seq dataset. Based on the calculated TIIC signature score of each LUAD cell, the LUAD cells were divided into two LUAD cell groups. ACTIVIN and IL‐17 signalling pathways were differentially active between the two LUAD cell groups (Figure S2A,B). The cell communication pattern between two LUAD cell groups and immune cells was also shown in Figure S2C. The TIIC signature score exhibited a strong association with multiple immunologic pathways (Figure S2D). Besides, several metabolic pathways, such as starch and sucrose metabolism, metabolism of lipids, and metabolism of carbohydrates, were found to be significantly associated with the TIIC signature score (Figure S2E,F).

### 3.4 Comparison of prognostic value between the TIIC signature score and previous signatures

**FIGURE 4:**
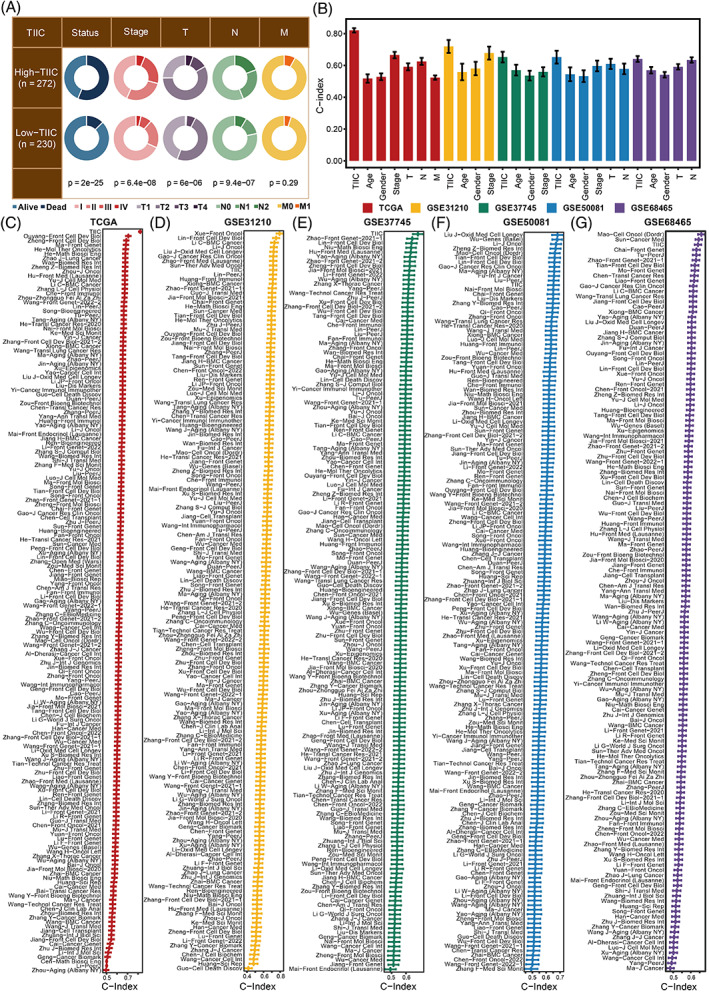
Prognostic value of the TIIC signature score. A. Circos plot of different clinical factors in two TIIC signature score groups. B. The C‐index of the TIIC signature score and various clinical factors in the TCGA, GSE31210, GSE37745, GSE50081, and GSE68465 datasets. C. The C‐index of the TIIC signature score and 168 LUAD models in the TCGA, GSE31210, GSE37745, GSE50081, and GSE68465 datasets.

The TIIC signature score was significantly associated with survival status, tumour stage, and TNM staging system in the TCGA dataset (Figure 4A). Besides, the TIIC signature score showed superior performance to age, gender, tumour stage, and TNM staging system regarding the C‐index in the TCGA dataset (Figure 4B). To further test the prognostic performance of the TIIC signature score, we incorporated 168 signatures and compared the C‐index in the TCGA, GSE31210, GSE37745, GSE50081, and GSE68465 datasets (Figure 4C–G). These 168 signatures exhibited associations with various biological features. Our TIIC signature displayed better performance than most other published signatures in the TCGA, GSE31210, GSE37745, GSE50081, and GSE68465 datasets.

### 3.5 Prediction of biological mechanisms related to the TIIC signature score

**FIGURE 5:**
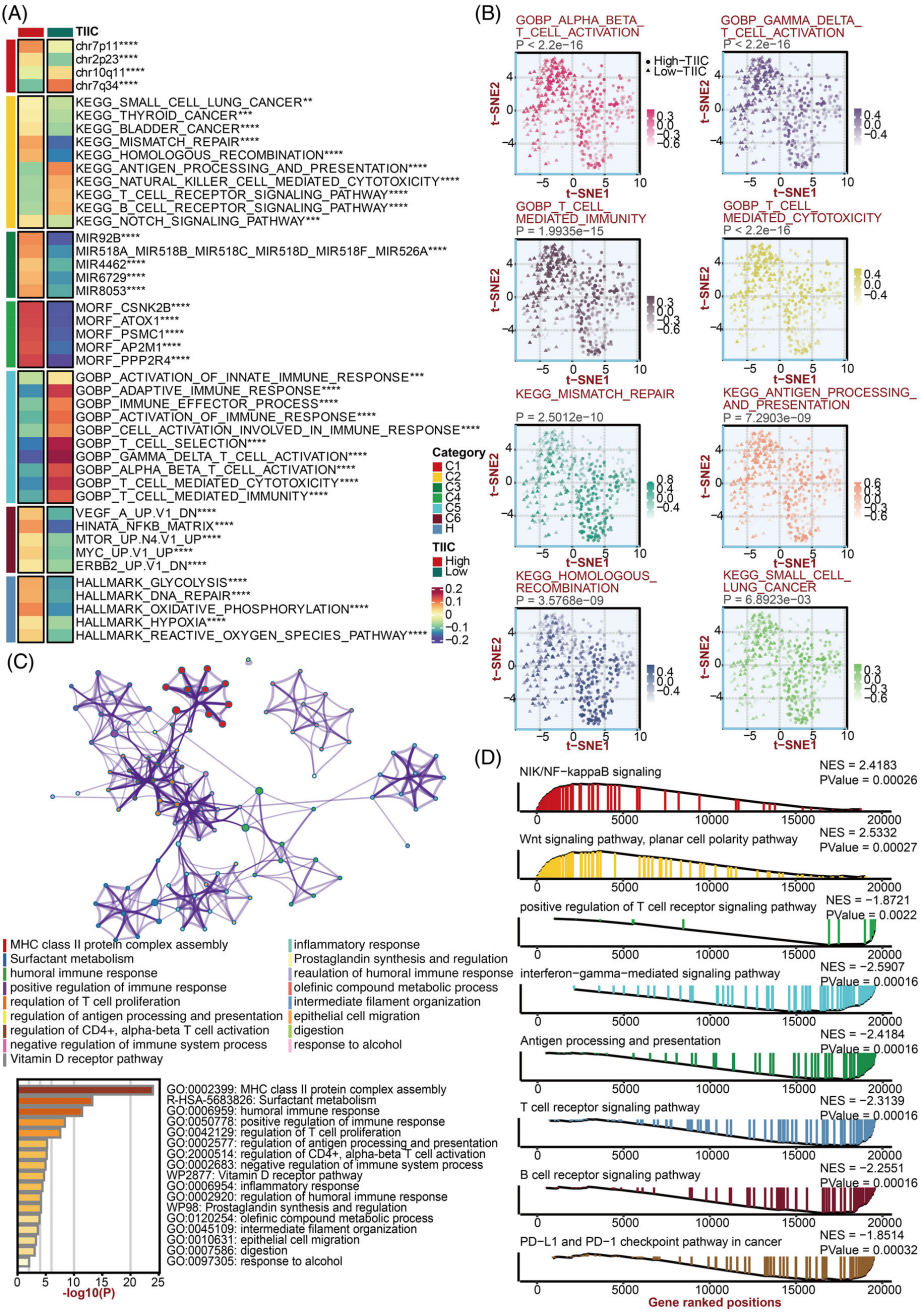
Biological peculiarities of the TIIC signature score in the TCGA dataset. A. MsigDB‐based GSVA analysis delineated the biological attributes of two TIIC signature score groups. B. t‐SNE plot of GO and KEGG terms delineated the differences in pathway activity in two TIIC signature score groups. C. Metascape‐based enrichment analysis of differentially expressed genes between two TIIC signature score groups. D. GSEA of GO and KEGG terms for the TIIC signature score.

Given the upregulated immune‐related characteristics displayed in the low‐score group, we tended to dig into the underlying biological mechanisms. The TIIC signature score exhibited a strong positive association with multiple tumorigenic pathways, including mismatch repair, homologous recombination, hypoxia, reactive oxygen species pathway, and small cell lung cancer (Figure 5A). In addition, the TIIC signature score exhibited a strong negative association with multiple immunologic pathways, including the B cell receptor signalling pathway, T cell receptor signalling pathway, T cell‐mediated immunity, activation of immune response, and antigen processing and presentation (Figure 5A). Significant differences in the tumorigenic and immunologic pathways in two TIIC signature score groups were further proved (Figure 5B). The DEGs between two TIIC signature score groups were enriched in immune infiltration and activation pathways (Figure 5C). In GSEA of GO and KEGG terms, the low TIIC signature group showed enrichment of the T cell receptor signalling pathway, antigen processing and presentation, IFN‐γ production, and B cell receptor signalling pathway as expected (Figure 5D). The high TIIC signature group showed enrichment of the NF‐κB signalling pathway and Wnt signalling pathway, planar cell polarity pathway, which was consistent with the overall survival analysis mentioned above (Figure 5D). Furthermore, LUAD patients with high expression of seven signature genes showed enrichment of the NF‐κB signalling pathway and Wnt signalling pathway, planar cell polarity pathway (Figure S3). On the one hand, this finding indicated that TIIC‐related PFN1, CFL1, HSP90AA1, PABPC1, RPS15, HMGB1, and CCDC85B could potentially activate NF‐κB and Wnt pathways. On the other hand, this finding further proved the stability and reliability of the signature. Taken together, our results revealed that a low TIIC signature represented a potency of superior immune response under immunotherapy.

### 3.6 TIIC signature displayed a substantial correlation with immune‐related characteristics

**FIGURE 6:**
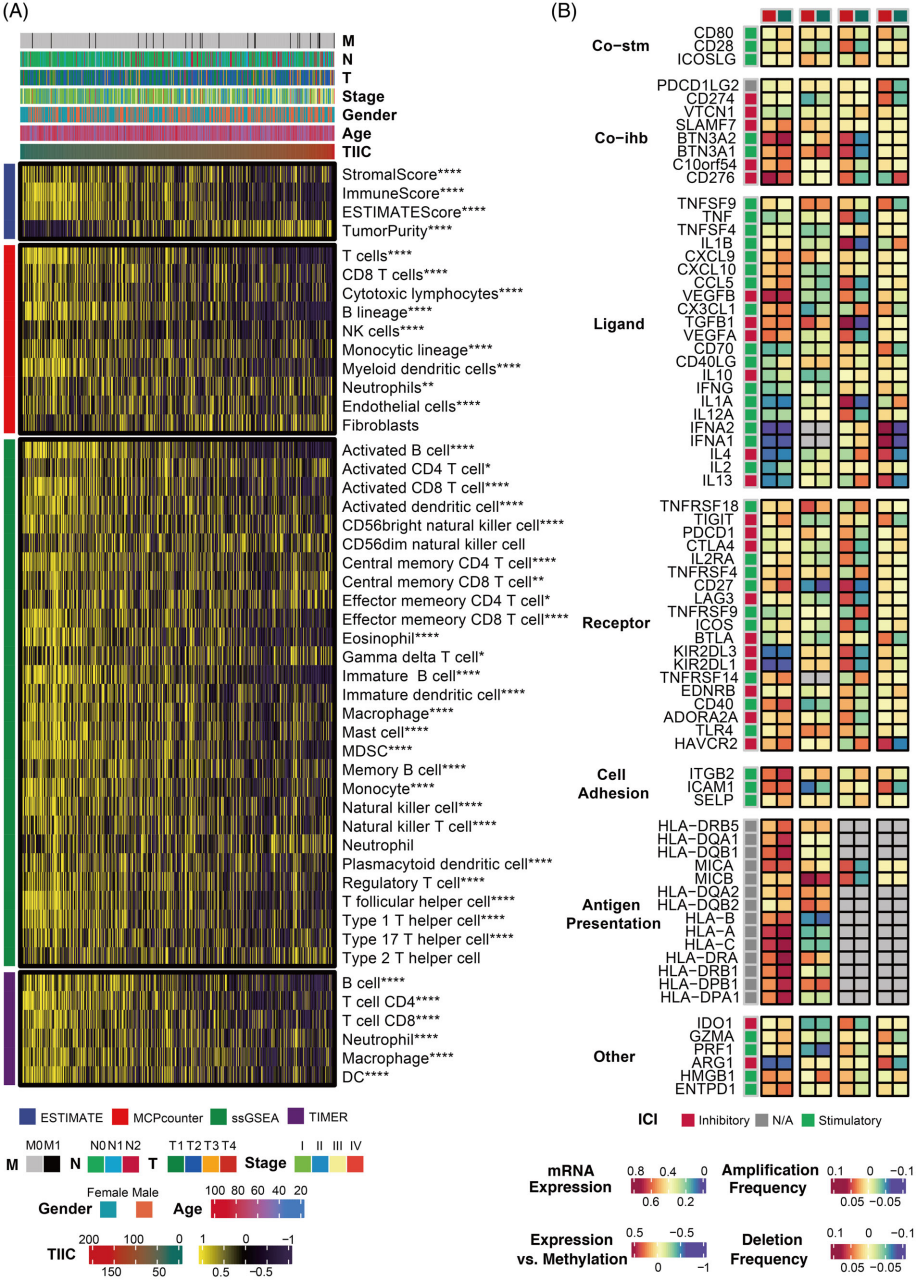
Immune characteristics of the TIIC signature score in the TCGA dataset. A. The correlation between the TIIC signature score and immune infiltrating cells. B. The correlation between the TIIC signature score and immune modulators.

To explore the immune status reflected by the TIIC signature score, we analysed the association between the TIIC signature score and immune infiltrating cells as well as immune checkpoints. As shown in Figure 6A,B, the low TIIC signature score group presented a higher level of immune infiltrating cells and immune modulators in the TCGA dataset, indicating an inflamed but relatively immune‐promoting microenvironment, which is the potential beneficiary of immunotherapy.^51^ Furthermore, we compared the status of APM score, CYT, GEP, IFN‐γ, and stromal fraction, TCR Shannon, and TCR Richness, which were related to a more immunoreactive microenvironment between the two TIIC signature score groups. It turned out they were all at a higher level in the low‐score group in the TCGA dataset (Figure S4A–G). Accordingly, a high TIIC signature score correlated with a higher level of TGF‐β, a critical marker for immune evasion (Figure S4H). The differences between six immune subtypes in two TIIC signature score groups are shown in Figure S4I, in which a high TIIC signature score correlated more with the wound healing subtype. Furthermore, TME‐related signatures were significantly upregulated in the low‐score group shown in the immunogram radar plot in the TCGA dataset (Figure S4J). Representative steps involved in the cancer immunity cycle, including the release of antigens, priming, and activation, immune cell recruitment and infiltration, recognition of cancer cells, and killing of cancer cells, were analysed and found to be more active in low score group in the TCGA dataset (Figure S4K).

### 3.7 Validation of predictive value of immunotherapy response of the TIIC signature score in multiple datasets

**FIGURE 7:**
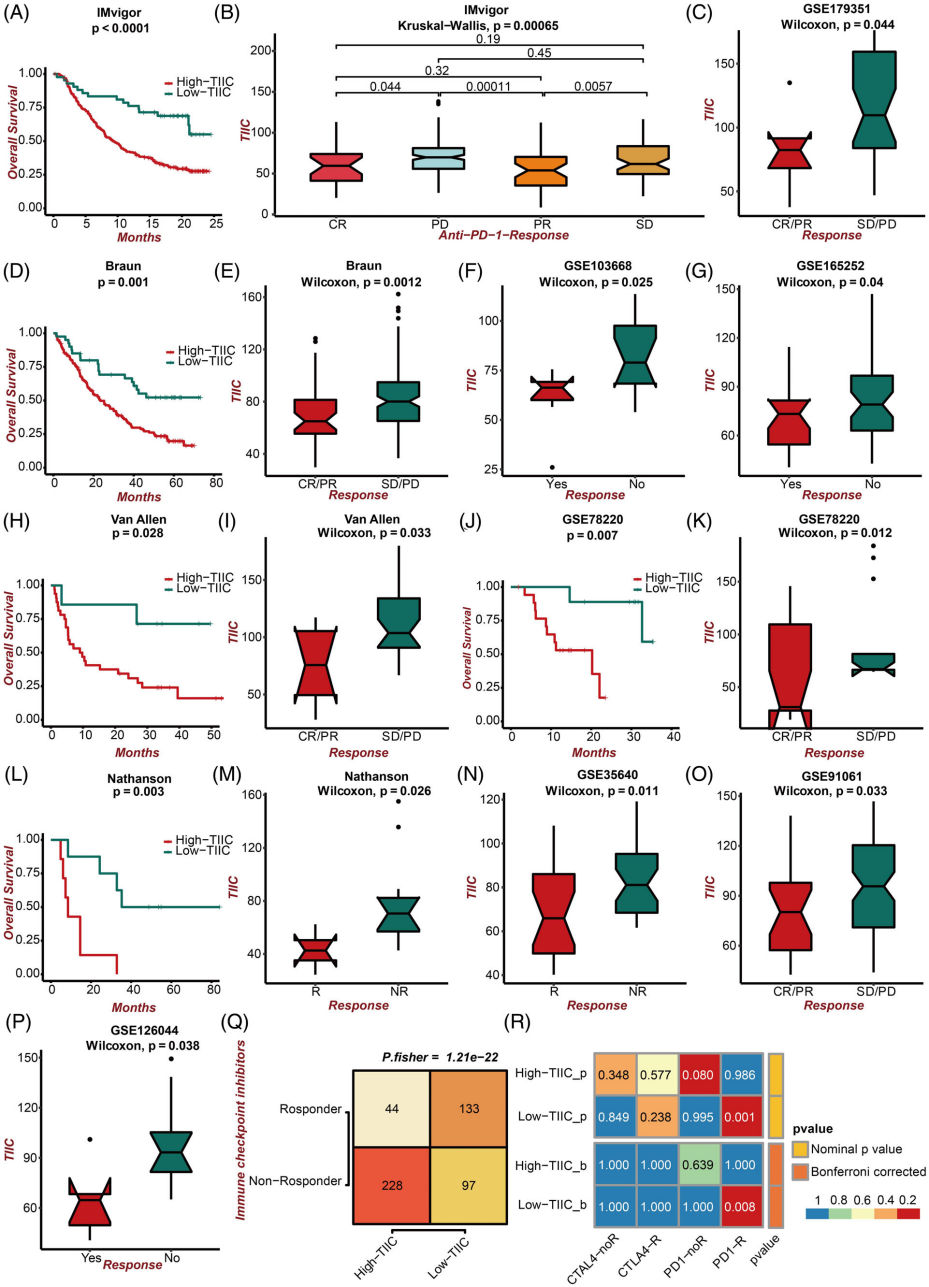
Immunotherapy response prediction of the TIIC signature score. A. Kaplan–Meier survival curves of the TIIC signature score regarding OS in the IMvigor dataset. B. The association between the IMvigor dataset's TIIC signature score and immunotherapy responses. C. The association between the GSE179351 dataset's TIIC signature score and immunotherapy responses. D. Kaplan–Meier survival curves of the TIIC signature score regarding OS in the Braun dataset. E. The association between the Braun dataset's TIIC signature score and immunotherapy responses. F. The association between the GSE103668 dataset's TIIC signature score and targeted therapy responses. G. The association between the GSE165252 dataset's TIIC signature score and immunotherapy responses. H. Kaplan–Meier survival curves of the TIIC signature score regarding OS in the Van Allen dataset. I. The association between the Van Allen dataset's TIIC signature score and immunotherapy responses. J. Kaplan–Meier survival curves of the TIIC signature score regarding OS in the GSE78220 dataset. K. The association between the GSE78220 dataset's TIIC signature score and immunotherapy responses. L. Kaplan–Meier survival curves of the TIIC signature score regarding OS in the Nathanson dataset. M. The association between the Nathanson dataset's TIIC signature score and immunotherapy responses. N. The association between the GSE35640 dataset's TIIC signature score and immunotherapy responses. O. The association between the GSE91061 dataset's TIIC signature score and immunotherapy responses. P. The association between the GSE126044 dataset's TIIC signature score and immunotherapy responses. Q. The TIDE algorithm predicted the association between the TIIC signature score and immunotherapy responses in the TCGA dataset. R. The association between the TIIC signature score and immunotherapy responses (anti‐PD‐1 and anti‐CTLA‐4) was predicted by the submap analysis in the TCGA dataset.

Given the predictive power of the TIIC signature score for immunotherapy benefit, we next verified the efficiency in multiple immunotherapy datasets. In the IMvigor dataset, UC patients with low TIIC signature scores presented better survival outcomes (Figure 7A). UC patients with low TIIC signature scores presented better responses to anti‐PD‐L1 immunotherapy (Figure 7B). Besides, in the Braun dataset, RCC patients with low TIIC signature scores showed better survival outcomes (Figure 7D). RCC patients with low TIIC signature scores presented better responses to anti‐PD‐1 immunotherapy (Figure 7E). Moreover, patients in the GSE179351 (COAD and PAAD) (Figure 7C) and GSE165252 (ESCA) (Figure 7G) datasets with low TIIC signature scores presented better responses to immunotherapy. Notably, patients in the GSE103668 (TNBC) dataset with low TIIC signature scores presented better responses to targeted therapy (Figure 7F). Additionally, in the Allen dataset, melanoma patients with low TIIC signature scores showed better survival outcomes (Figure 7H). Melanoma patients with low TIIC signature scores presented better responses to anti‐CTLA‐4 immunotherapy (Figure 7I). In the GSE78220 dataset, melanoma patients with low TIIC signature scores showed better survival outcomes (Figure 7J). Melanoma patients with low TIIC signature scores presented better responses to anti‐PD‐1 immunotherapy (Figure 7K). In the Nathanson dataset, melanoma patients with low TIIC signature scores showed better survival outcomes (Figure 7L), and melanoma patients with low TIIC signature scores presented better responses to anti‐CTLA‐4 immunotherapy (Figure 7M). Patients in the GSE35640 (melanoma) (Figure 7N) and GSE91061 (melanoma) (Figure 7O) datasets with low TIIC signature scores also presented better responses to immunotherapy. Notably, patients in the GSE126044 (NSCLC) dataset with low TIIC signature scores presented better responses to immunotherapy (Figure 7P). The TIDE algorithm revealed that a low TIIC signature score was significantly associated with better immunotherapy responses in the TCGA dataset (Figure 7Q). The submap analysis also revealed that a low TIIC signature score was significantly associated with better anti‐CTLA‐4 and anti‐PD‐1 immunotherapy responses in the TCGA dataset (Figure 7R).

### 3.8 Prediction of metabolic characteristics related to the TIIC signature score

**FIGURE 8:**
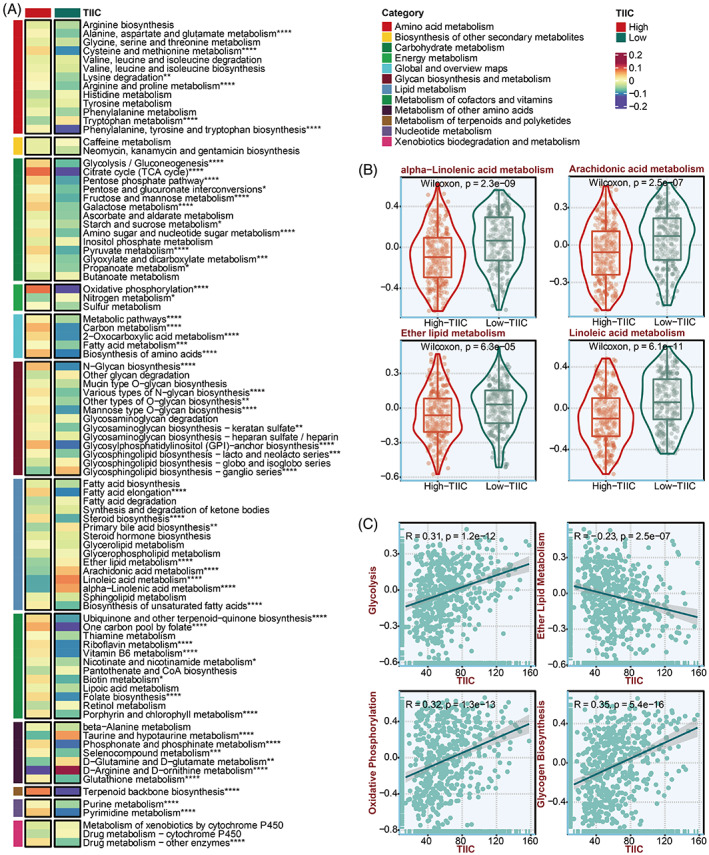
Metabolic characteristics of the TIIC signature score in the TCGA dataset. A. KEGG‐based GSVA analysis of the metabolic pathways of 12 metabolic categories in two TIIC signature score groups. B. The differences of lipid metabolism pathways in two TIIC signature score groups. C. The correlation between the TIIC signature score and literature‐based GSVA analysis of metabolic pathways.

To investigate an extensive spectrum of metabolic reprogramming in two TIIC signature score groups, GSVA was executed against metabolic pathways from the KEGG database. The TIIC signature score was also positively correlated with many metabolic pathways facilitating tumour growth, such as carbohydrate metabolism, glycan biosynthesis and metabolism, and the metabolism of cofactors and vitamins (Figure 8A). Notably, the lipid metabolism pathways, previously proved to promote anti‐tumour immune response,^52^ were significantly more activated in the low TIIC signature score group (Figure 8B). In addition, GSVA was executed against metabolic pathways from the previous literature, in which the TIIC signature score was negatively associated with ether lipid metabolism and positively associated with glycolysis, oxidative phosphorylation, and glycogen biosynthesis (Figure 8C).

### 3.9 Multi‐omics alteration characteristics related to the TIIC signature score

**FIGURE 9:**
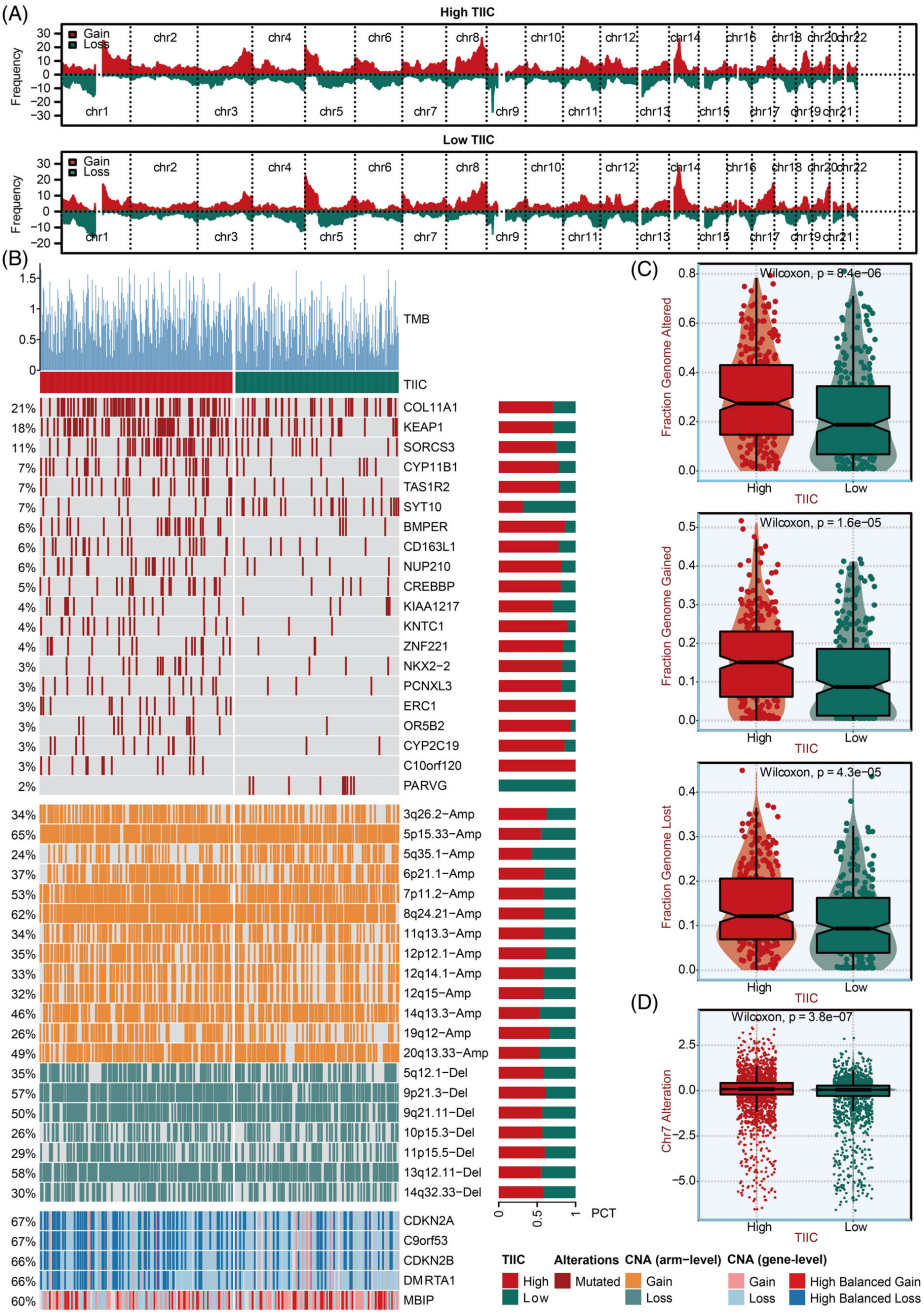
Multi‐omics alteration characteristics of the TIIC signature score in the TCGA dataset. A. GISTIC 2.0‐based chromosome amplifications and deletions in two TIIC signature score groups. B. Genomic alteration landscape in two TIIC signature score groups. C. The fraction of genome alteration, the fraction of genome gained, and the fraction of genome lost in two TIIC signature score groups. D. The distribution of Ch7 alterations in two TIIC signature score groups.

The different frequently altered chromosomes were observed in two TIIC signature score groups (Figure 9A). The specific altered genomic regions are shown in Figure 9B. The high TIIC signature score group presented high chromosomal instability, featured by FGA, FGG, and FGL (Figure 9C). We also observed that the amplification of Chr7, represented by EGFR mutation (7p11.2), was particularly evident for the high TIIC signature score group (Figure 9D).

Subsequently, the genomic alterations of the TIIC signature score were explored. TP53, TTN, MUC16, CSMD3, and RYR2 were the top five most frequently mutated genes in the high TIIC signature score group (Figure S5A). The most differentially mutated genes between the two TIIC signature score groups are depicted (Figure S5B). Notably, the mutation rates of COL11A1 and KEAP1 were extraordinarily high in the high TIIC signature score group (Figure S5B). Accordingly, LUAD patients with high COL11A1 and KEAP1 expression presented worse survival outcomes (Figure S6A). COL11A1‐mutated LUAD patients and KEAP1‐mutated LUAD patients had significantly higher COL11A1 and KEAP1 expression, respectively (Figure S6B). The co‐occurrence and mutually exclusive mutations between two TIIC signature score groups are displayed (Figure S5D).

### 3.10 Prediction of drug response related to the TIIC signature score

Acetalax, Afatinib, AZD3759, Gefitinib, Ibrutinib, Lapatinib, Osimertinib, and Sapitinib were found with significantly better drug sensitivity in the high TIIC signature score group based on the GDSC database (Figure S7A). The less the CMap score is, the more likely the drug is to reverse the molecular features of the disease based on the theory of CMap. Notably, butein was found with the lowest CMap score, most likely to cure the LUAD patients with a high TIIC signature score (Figure S7B). Furthermore, CTRP‐based SB‐743921 (Figure S7C) and PRISM‐based ispinesib (Figure S7D) had the highest correlation with the TIIC signature score. These two drugs were found with significantly better drug sensitivity in the high TIIC signature score group.

### 3.11 Validation of prognostic value of the TIIC signature score in the LUAD tissue array

**FIGURE 10:**
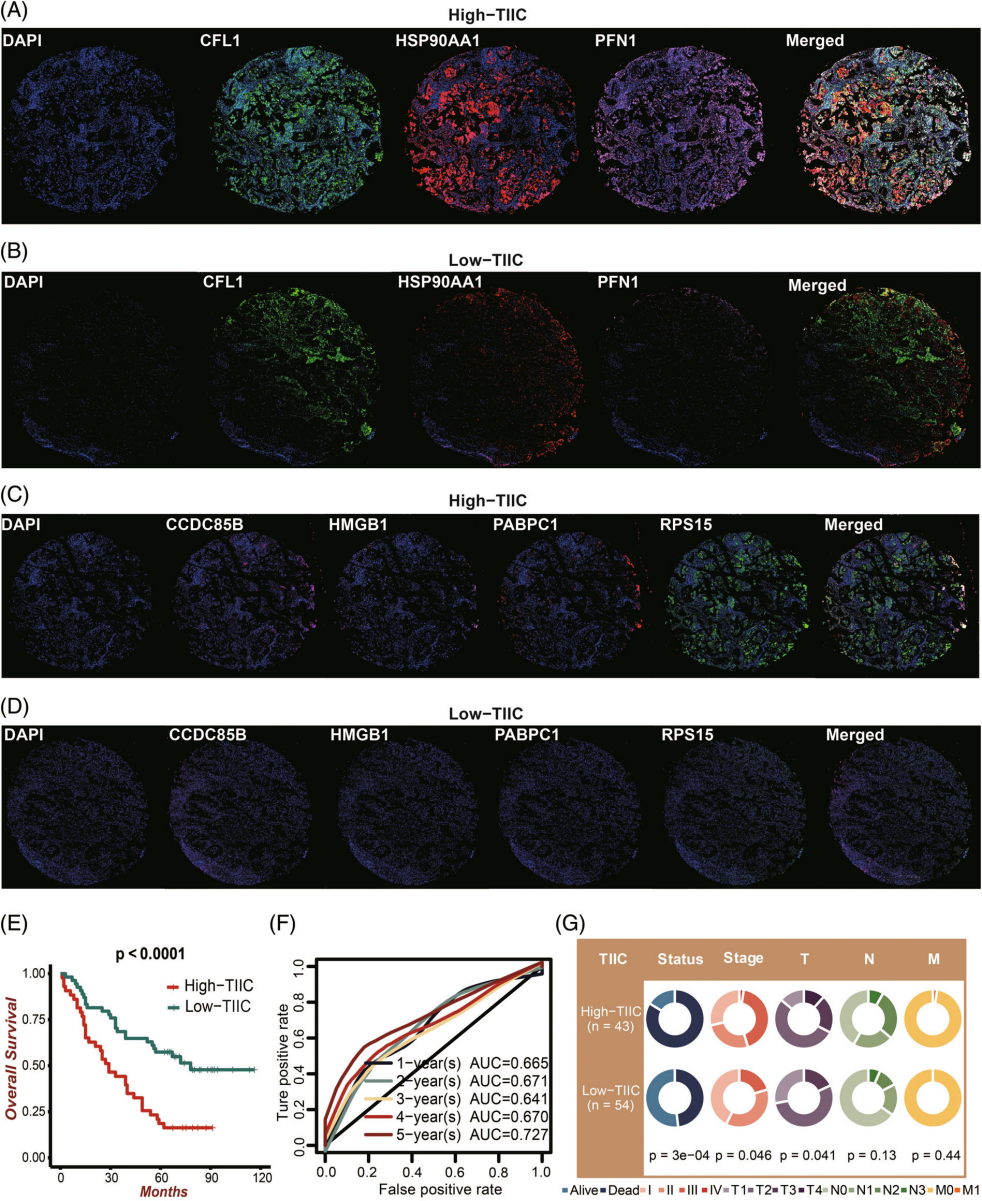
Prognostic value of the TIIC signature score in the LUAD tissue array. A. Representative images of CFL1, HSP90AA1, and PFN1 based on multiplex IF staining in the high TIIC signature score group. B. Representative images of CFL1, HSP90AA1, and PFN1 based on multiplex IF staining in the low TIIC signature score group. C. Representative images of CCDC85B, HMGB1, PABPC1, and RPS15 based on multiplex IF staining in the high TIIC signature score group. D. Representative images of CCDC85B, HMGB1, PABPC1, and RPS15 based on multiplex IF staining in the low TIIC signature score group. E. Kaplan–Meier survival curves of the TIIC signature score regarding OS. F. Time‐dependent ROC curves of the TIIC signature score regarding 1‐, 2‐, 3‐, 4‐ and 5‐year OS. G. Circos plot of different clinical factors in two TIIC signature score groups.

The multiplex immunofluorescence (IF) staining of PFN1, CFL1, HSP90AA1, PABPC1, RPS15, HMGB1, and CCDC85B was performed in the LUAD tissue array. Based on the quantified staining intensity of the seven TIIC‐RNAs, the TIIC signature score was developed according to the previous procedure. Representative images of the seven TIIC‐RNAs in the two TIIC signature score groups were selected for exhibition (Figure 10A–D). In accordance with the earlier findings, LUAD patients with low TIIC signature scores showed better survival outcomes (Figure 10E). The time‐dependent ROC curves quantified by AUC values at 1, 2, 3, 4, and 5 years of OS proved the predictive value of the TIIC signature score in prognosis (Figure 10F). The TIIC signature score was also significantly associated with survival status, tumour stage, and T staging system in the TCGA dataset (Figure 10G).

## 4 DISCUSSION

With the fast development of sequencing technology at the bulk and single‐cell levels, numerous tumour markers and targets have been identified, which deepens the understanding of the tumorigenic process. Accordingly, increasing numbers of diagnostic and prognostic signatures have been constructed to predict cancer patients' clinical outcomes and treatment efficacy. TIICs, mainly consisting of T cells, B cells, mast cells, NK cells, and DCs, commonly exist in the TME of almost all solid tumours. TIICs have been generally believed to impact the proliferation, migration, and invasion of tumours.^53^ More importantly, as an emerging star in tumour treatment, TIICs are proven to, directly and indirectly, enhance immunotherapy efficacy by reinvigorating an efficacious antitumoral immune response.^54^ Given that, the emergence of novel high‐throughput sequencing methods has also allowed the identification of many TIIC‐based signatures. However, these signatures are mainly based on the TIICs calculated from bulk sequencing data, which could not accurately reflect the TME. Many of these signatures also show limited value in clinical applications since they fail to deliver robust and reliable performance in the validation dataset. Notably, recent applications of scRNA‐seq in dissecting the TME have brought important insights into the biology of TIICs, including their dynamics, heterogeneity, potential pathogenic roles, and response to immunotherapy.^55^ Besides, ML has been a widely applied method for excavating the most valuable data in the big data era. Therefore, this study first analysed the scRNA‐seq data of LUAD for precise identification of the TIICs. Subsequently, a powerful TIIC signature based on 26 ML algorithms was developed based on bulk sequencing data.

Starting from TIICs identified from the scRNA‐seq data, we utilized a novel computational framework to screen out immune‐related RNAs. And TSI score was calculated to determine LUAD TIIC‐RNAs based on different expression levels between immune cells and LUAD cells. Subsequently, we established a TIIC signature including seven TIIC‐RNAs (PFN1, CFL1, HSP90AA1, PABPC1, RPS15, HMGB1, and CCDC85B) based on 26 ML algorithms. A higher score of this signature was associated with inferior overall survival, resulting from activated tumorigenic pathways such as NOTCH, WNT, JAK–STAT, and NF‐κB signalling pathways. The TIIC signature score also showed a higher prognostic value than age, gender, and TNM staging system. To prove the predictive efficiency of our TIIC signature, we compared it with 168 published signatures in the TCGA, GSE31210, GSE37745, GSE50081, and GSE68465 datasets. It turned out that the TIIC signature score we established possessed the most potent potency to predict prognosis. Besides, the TIIC signature score was calculated based on the expression levels of PFN1, CFL1, HSP90AA1, PABPC1, RPS15, HMGB1, and CCDC85B by IF staining. The remarkable performance in predicting survival outcomes indicated that the TIIC signature score might be of great use in the future.

It should be noted that immunotherapy is determined by several factors, including infiltration of immune cells, IFN‐γ production, TGF‐β level, and immune modulators.^51^, ^56^ The low TIIC signature score group showed a higher level of immune infiltrating cells, immune modulators, and biomarkers representing the immunoreactive microenvironment (CYT, GEP, TCR, and IFN‐γ) in the TCGA dataset. Moreover, a lower TIIC signature score was correlated with a more active cancer immunity cycle and multiple immunologic pathways, demonstrating its decent value in predicting immunotherapy response. The validation performed in multiple immunotherapy datasets proved the predictive efficiency subsequently. In the IMvigor (UC), GSE91061 (melanoma), GSE35640 (melanoma), GSE78220 (melanoma), Van Allen (melanoma), Nathanson (melanoma), GSE179351 (COAD and PAAD), Braun (renal cell carcinoma), GSE165252 (ESCA), and GSE126044 (NSCLC) datasets, the low TIIC signature score group exhibited superior immunotherapy response and overall survival. Surprisingly, the low TIIC signature score group predicted better chemotherapy response in the GSE103668 (TNBC) dataset.

The TIIC signature score we established in this study exhibited abundant immune‐related characteristics and strong associations with prognosis, indicating its vast potential in clinical practice complementary to current biomarkers. Notably, the TIIC signature score outperformed 168 previously established models. Despite the potential value of the TIIC signature score, many questions remain to be resolved. All datasets included in this study were single‐centred and retrospective; future validation should be performed in prospective multi‐centred datasets. In addition, the mechanisms by which the seven signature genes regulate TME and immune response are complex. PFN1 induced tumour metastasis by promoting microvesicle secretion in non‐small cell lung cancer,^57^ and increased expression of PFN1 was found in DCs.^58^ RHOA/CDC42‐CFL1 axis is critical in mediating the tumour cell migration in lung cancer.^59^ Direct targeting of HSP90AA1 with daurisoline could destabilize β‐catenin to suppress lung cancer tumorigenesis.^60^ Notably, HSP90AA1 functions in antigen presentation, immune effector cell tasks, and regulation of inflammatory processes.^61^ E3 ligase MKRN3 is a tumour suppressor regulating PABPC1 ubiquitination in non‐small cell lung cancer,^62^ and PABPC1 signalling controlled the secretion of miR‐19a‐3p by CD8 T cells.^63^ Cancer‐cell‐secreted CXCL11 could promote CD8 T cell infiltration through the docetaxel‐induced release of HMGB1 in NSCLC.^64^ CCDC85B also promotes non‐small cell lung cancer cell proliferation and invasion.^65^ The interaction between these TIIC‐RNAs and immune microenvironment components is unknown and needs to be investigated in‐depth, which may help develop potential therapeutic targets and benefit future applications.

However, there are also limitations to this study. First, the specific biological functions and mechanisms of the seven TIIC‐RNAs in the activity of immune cells need to be further verified in vitro and in vivo. Second, due to the complexity of the tissue, scRNA‐seq may not define the immune cells and LUAD cells with 100% accuracy, which might lead to some inconsistency in the expression patterns of the seven TIIC‐RNAs. Third, a multi‐centred cohort is expected to further validate the TIIC signature score's prognostic value. Forth, more immunotherapy datasets of LUAD are needed to validate the potential of the TIIC signature score in predicting immunotherapy response.

All in all, by holistic analysis of bulk transcriptome data and single‐cell sequencing data of immune cells and LUAD cells based on the novel computational framework and 26 ML algorithms, the TIIC signature score with enormous potency was established to distinguish the outcome of LUAD patients and predict the response of immunotherapy. As newly demonstrated predictive biomarkers, the TIIC signature score enables more precise identification of the LUAD patients who benefit from immunotherapy and should be validated and applied shortly.

## AUTHOR CONTRIBUTIONS

Hao Zhang, Quan Cheng, Nan Zhang, Zaoqu Liu, and Xizhe Li designed and drafted the manuscript. Hao Zhang, Quan Cheng, Nan Zhang, Shuyu Li, Jian Zhang, Peng Luo, Ran Zhou, Wantao Wu, Zeyu Wang, Jie Wen, Xisong Liang, Xun Zhang, Bo Zhang, Sirui Ouyang, and Ziyu Dai wrote figure legends and revised the manuscript. Xisong Liang, Jie Wen, Xun Zhang, and Bo Zhang performed data collection. Nan Zhang conducted data analysis. Hao Zhang conducted the experiments. All authors have read and approved the final manuscript.

## FUNDING INFORMATION

Financial support was provided by the National Natural Science Foundation of China (Nos. 82073893, 82203833). Hunan Provincial Natural Science Foundation of China (Nos. 2022JJ20095, 2022JJ40798). Hunan Science and Technology Innovation Talent Program Excellent Postdoctoral Innovation Talent Project (No. 2021RC2029). Hunan Provincial Health Committee Foundation of China (No. 202204044869).

## CONFLICT OF INTEREST STATEMENT

All authors declare that they have no competing interests.

## ETHICS STATEMENT

The ethics of the tissue microarray was approved.

## Supporting information


**Appendix S1:** Supporting information

## Data Availability

All data used in this work can be acquired from the Gene Expression Omnibus (GEO; https://www.ncbi.nlm.nih.gov/geo/), the Cancer Genome Atlas (TCGA) datasets (https://xenabrowser.net/), and the European Nucleotide Archive (ENA, https://www.ebi.ac.uk/ena/).
