# Supplementary material for: An artificial intelligence network‐guided signature for predicting outcome and immunotherapy response in lung adenocarcinoma patients based on 26 machine learning algorithms
Source: Cell Prolif. 2023 Feb 23;56(4):e13409. doi: 10.1111/cpr.13409 (PMC10068958; doi:10.1111/cpr.13409)
Supplement: Supplementary file 1 — Appendix S1: Supporting information [file CPR-56-e13409-s001.docx]

Materials and Methods

Development of tumor-infiltrating immune cell-related signature

A computational framework based on several algorithms was developed for identifying the TIIC signature score by integrative analysis of immune cells and LUAD cells at the scRNA sequencing level and LUAD cancer tissues at a bulk level as follows:

1. The top 15% of RNAs, according to the expression values, were determined as potential immune-related RNAs.
2. The tissue specificity index (TSI)^19^ was used to determine the potential immune-related RNAs:

TSI_RNA_ = $\frac{\sum_{i=1}^{N} (1-x_{RNA,i})}{N-1}$

where N refers to the number of immune cell types and x_RNA,i_ refers to the expression intensity of immune cell i for RNA based on the normalized maximal expression value of RNA in each cell type. TSI has the range of 0 to 1, in which an immune cell-general RNA is defined when TSI equals 0, and an immune cell-specific RNA is defined when TSI equals 1. The RNAs with high expression in all immune cell types were classified as immune-related universal RNAs (iuRNA).

1. iuRNAs with significant upregulation in immune cell types and downregulation in LUAD cells were defined as TIIC-RNAs.
2. ML for classification, including Boruta, eXtreme gradient boosting (Xgboost), least absolute shrinkage and selection operator regularized logistic regression (LassoLR), support vector machine (SVM), random forest (RF), and prediction analysis for microarrays (Pamr), was further used to screen out the most valuable TIIC-RNAs.
3. Univariate Cox proportional hazards regression analysis was further applied to screen the candidate prognostic TIIC-RNAs.
4. Three ML algorithms for classification, including random survival forest (RSF), least absolute shrinkage and selection operator regularized cox regression (LassoCox), and cox model by likelihood-based boosting (CoxBoost), were further used to evaluate the importance of the prognostic TIIC-RNAs.
5. 20 ML algorithms for scoring, including RSF, conditional random forests (CForest), LassoCox, elastic net regression (Enet), Ridge, gradient boosting with regression trees (BlackBoost), regression for a parametric survival model (SurvReg), conditional inference trees (CTree), CoxPH, ObliqueRSF, StepwiseCox, SurvivalSVM, generalized boosted regression models (GBM), Ranger, partial least squares regression for cox models and related techniques (PlsRcox), gradient boosting with component-wise linear models (GlmBoost), supervised principal components (SuperPC), akritas conditional non-parametric survival estimator (Akritas), CoxBoost, and recursive partitioning and regression trees (Rpart), were used to determine the most reliable model based on comprehensive C-index.
6. The TIIC signature score was developed based on the prognostic TIIC-RNAs using the RSF algorithm. The survival trees were split using the log-rank score test^20^. The x-variable x was assumed to be ordered as x_1_ ≤ x_2_ ≤ … ≤ x_n_. The “ranks” for each survival time T_j_ (j ∈ [1, …, n]) were computed. The equation is as follows:

a_j_ = δ_j_ - $\sum_{k=1}^{{}_{j}} \frac{{}_{k}}{n- {}_{k} +1}$

where ${}_{k}$ = #[t : T_t_ ≤ T_k_] and ${}_{j}$ refers to the index of the order for T_j_. The log-rank score test is as follows:

The TIIC signature score = S (x, c) =$\frac{\sum_{xkc} (a_{j} -n_{l}\overline{a})}{\sqrt{n_{l}\left( 1- \frac{n_{l}}{n} \right)S_{a}^{2}}}$

where $\overline{a}$ and $s_{a}^{2}$ refer to the sample mean and sample variance of [a_j_ : j = 1, . . . , n], respectively.

Functional annotation of the TIIC signature score

Immune modulators and six immune subtypes were collected^21^. T cell-inflamed gene expression profile (GEP) and Cytotoxic activity (CYT) were calculated as previously described^22,23^. Antigen presentation machinery (APM) score, T cell receptor (TCR) Richness, TCR Shannon, transforming growth factor β (TGF-β), interferon-gamma (IFN-γ), and stromal fraction was also analyzed. The Tumor Immune Estimation Resource (TIMER) algorithm^24^ of six immune cells, the single-cell gene set enrichment analysis (ssGSEA) algorithm^25^ of 28 immune cells, Microenvironment Cell Populations-counter (MCPcounter) algorithm^26^ of ten immune cells, and Estimation of STromal and Immune cells in MAlignant Tumours using Expression data (ESTIMATE) algorithm^27^ were used for quantifying immune infiltrating cells. The cancer immunity cycle proposed by Karasaki was calculated^28,29^. TME signatures independently developed by Kobayashi^30^ and Bagaev^31^ were calculated using GSVA. GSVA and GSEA of gene ontology (GO) and KEGG terms were performed. Metascape was used for enrichment analysis^32^. 114 metabolic pathways from the previous literature were quantified using GSVA^33^. Metabolic pathways from the KEGG database were also quantified using GSVA.

Immunotherapeutic response prediction

The Van Allen (melanoma)^34^, Nathanson (melanoma)^35^, GSE35640 (melanoma)^36^, GSE91061 (melanoma)^37^, GSE78220 (melanoma)^38^, IMvigor210 (urothelial carcinoma, UC)^39^, Braun (renal cell carcinoma, RCC)^40^, GSE179351 (colorectal adenocarcinoma and pancreatic adenocarcinoma, COAD and PAAD)^41^, GSE165252 (esophageal adenocarcinoma, ESCA)^42^, GSE103668 (triple-negative breast cancer, TNBC)^43^, and GSE126044 (non-small cell lung cancer, NSCLC) datasets were collected, and the TIIC signature score was calculated in each dataset for prediction of immunotherapy response. The Subnetwork Mappings in Alignment of Pathways (Submap) analysis and Tumor Immune Dysfunction and Exclusion (TIDE) algorithm were also utilized in this section^44,45^.

Supplementary Figures


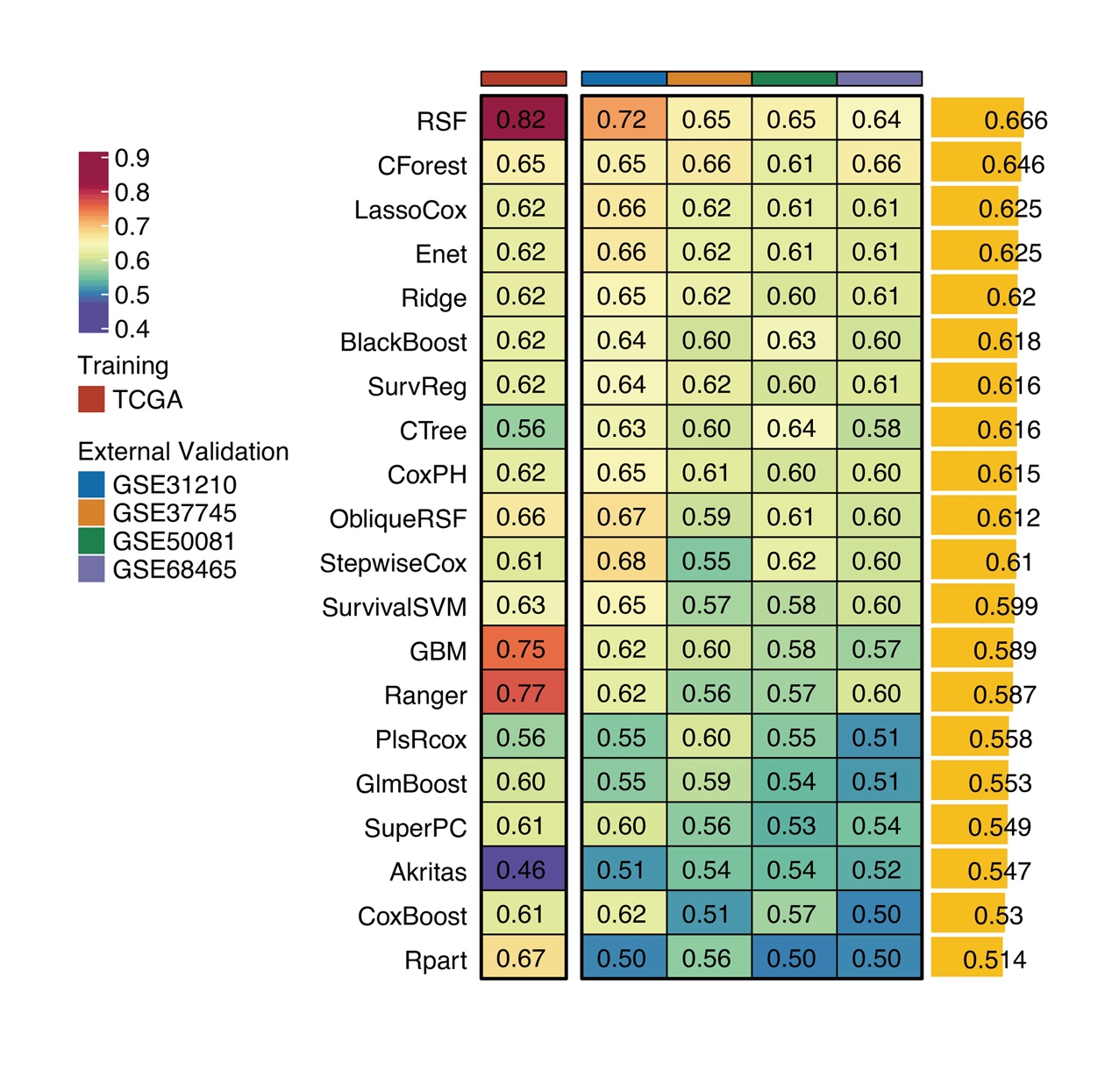


Figure S1. RSF was selected as the TIIC signature modeling method among 20 ML methods for scoring.


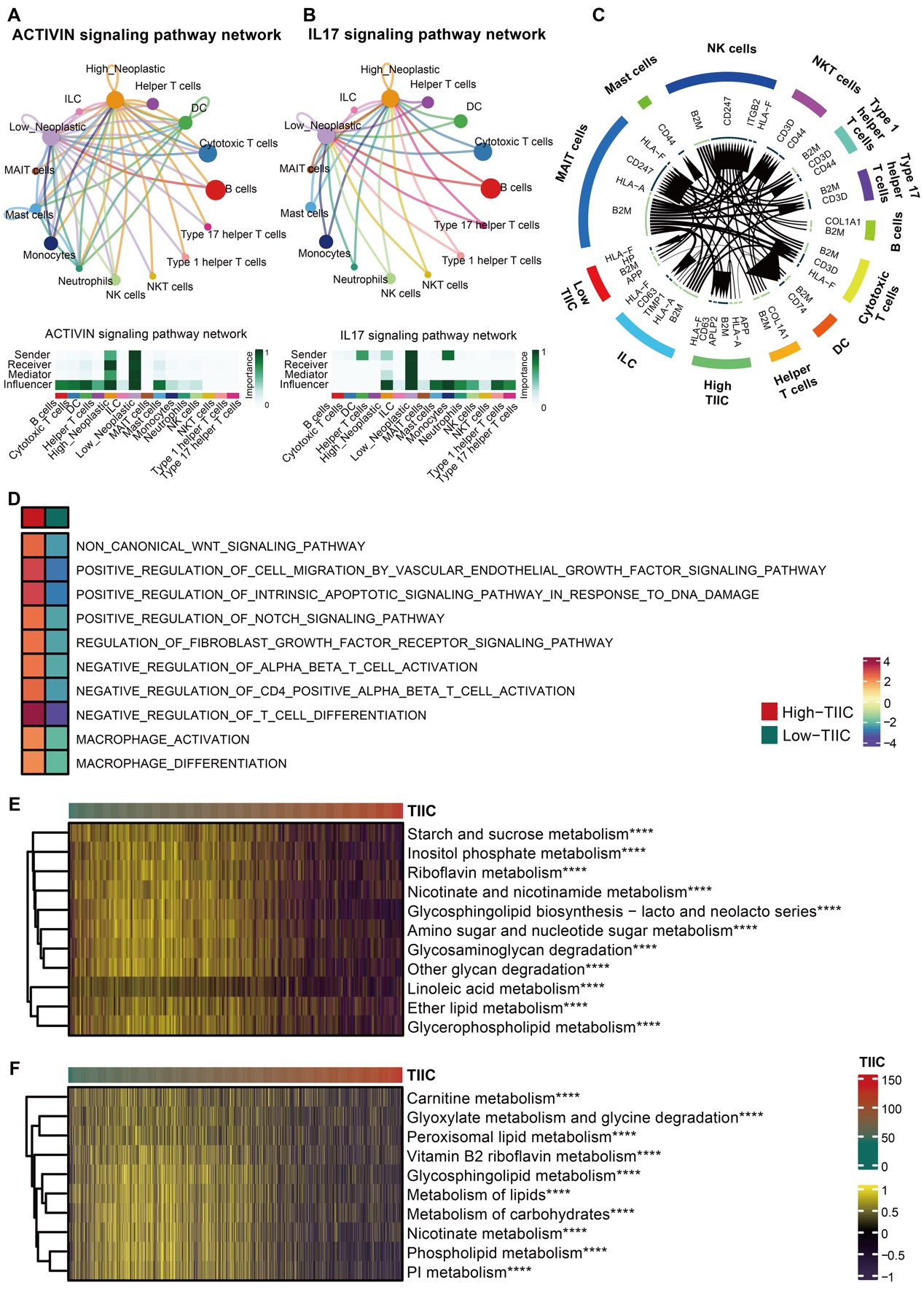


Figure S2. Functional annotation of the TIIC signature at the single-cell level. A. Cell communication among the identified cells regarding the ACTIVIN signaling pathway based on the R package cellchat. B. Cell communication among the identified cells regarding the IL-17 signaling pathway based on the R package cellchat. C. Cell communication among the identified cells based on the R package iTalk. D. GSVA of tumorigenic and immunogenic pathways between two TIIC signature groups. E. KEGG of metabolic pathways between two TIIC signature groups. F. REACTOME of metabolic pathways between two TIIC signature groups.


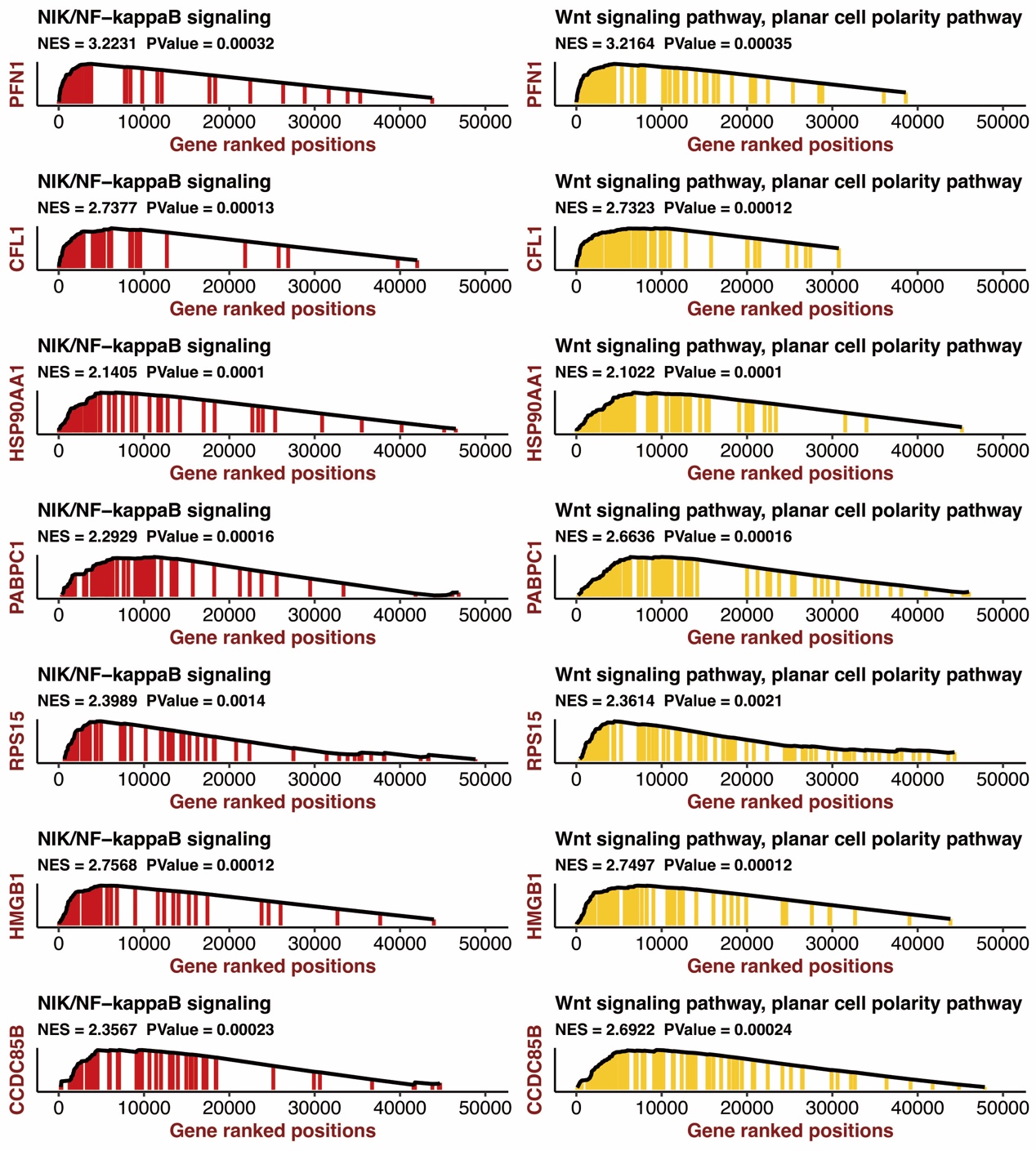


Figure S3. GSEA of NF-kappa B signaling pathway and Wnt signaling pathway, planar cell polarity pathway for the seven signature genes.


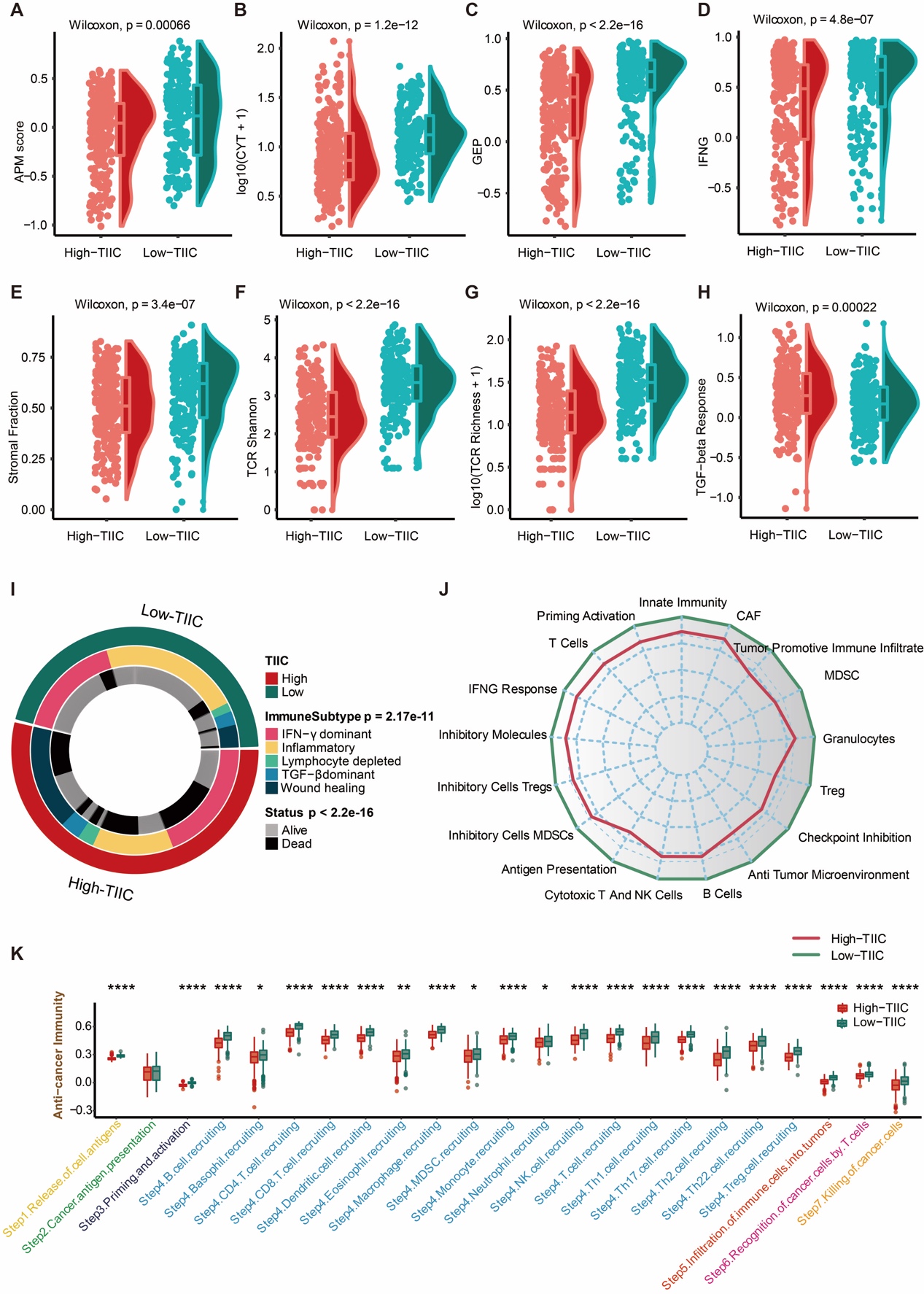


Figure S4. Relationship between the TIIC signature and immunotherapy predictors in the TCGA dataset. A. Box plot showing the levels of APM score between two TIIC signature score groups. B. Box plot showing the levels of CYT between two TIIC signature score groups. C. Box plot showing the levels of GEP between two TIIC signature score groups. D. Box plot showing the levels of IFN-γ between two TIIC signature score groups. E. Box plot showing the levels of stromal fraction between two TIIC signature score groups. F. Box plot showing the levels of TCR Shannon between two TIIC signature score groups. G. Box plot showing the levels of TCR Richness between two TIIC signature score groups. H. Box plot showing the levels of TGF-β response between two TIIC signature score groups. I. Circos plot showing the association between TIIC signature and five immune subtypes. J. The correlation between TIIC signature score and GSVA-based TME signatures developed by Bagaev and Kobayashi. K. The differences in cancer immunity cycle in two TIIC signature score groups.


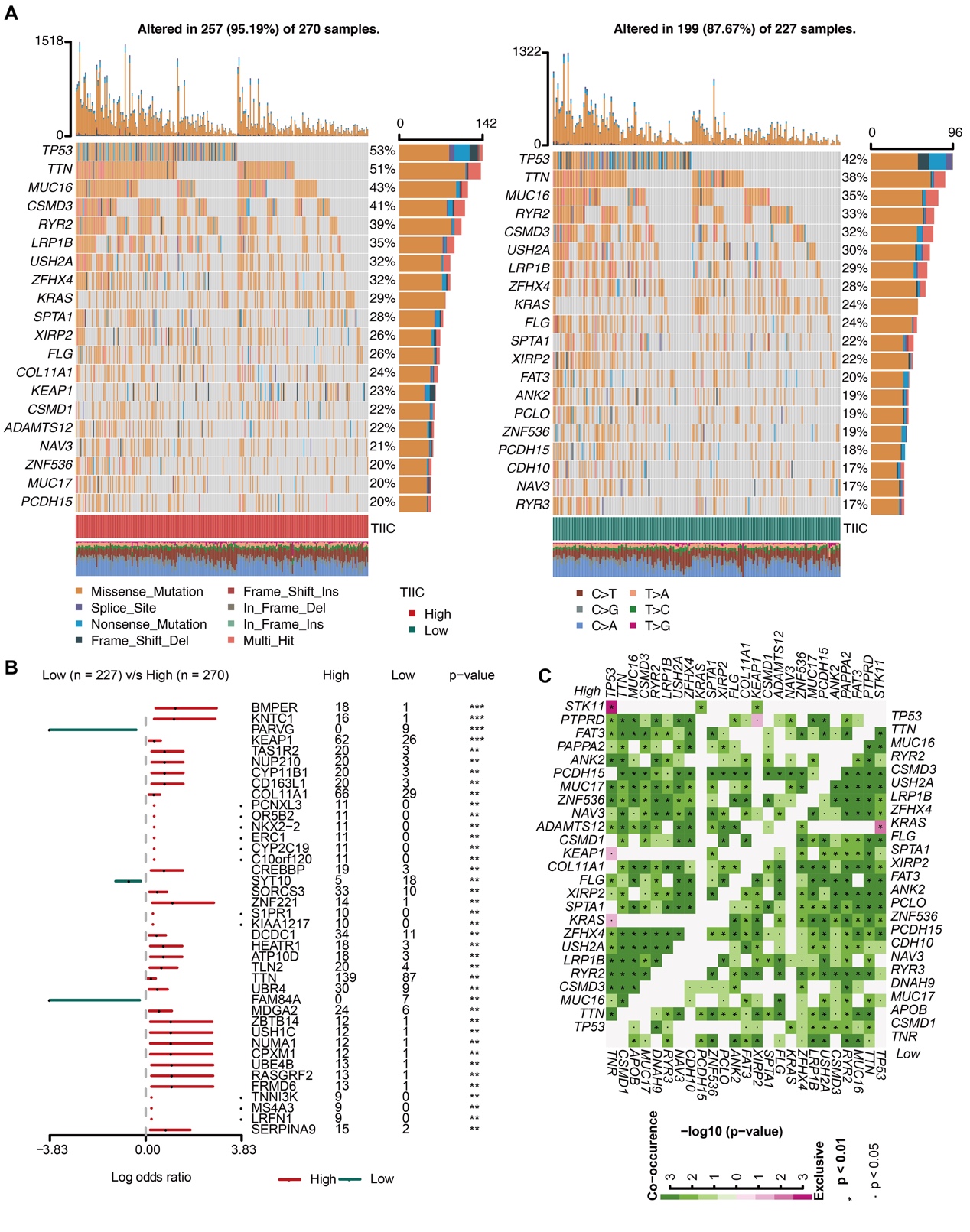


Figure S5. Genomic alterations of the TIIC signature. A. Oncoplots of the mutated genes in two TIIC signature score groups. B. The most differentially mutated genes between two TIIC signature score groups. C. The co-occurrence and mutually exclusive mutations between two TIIC signature score groups.


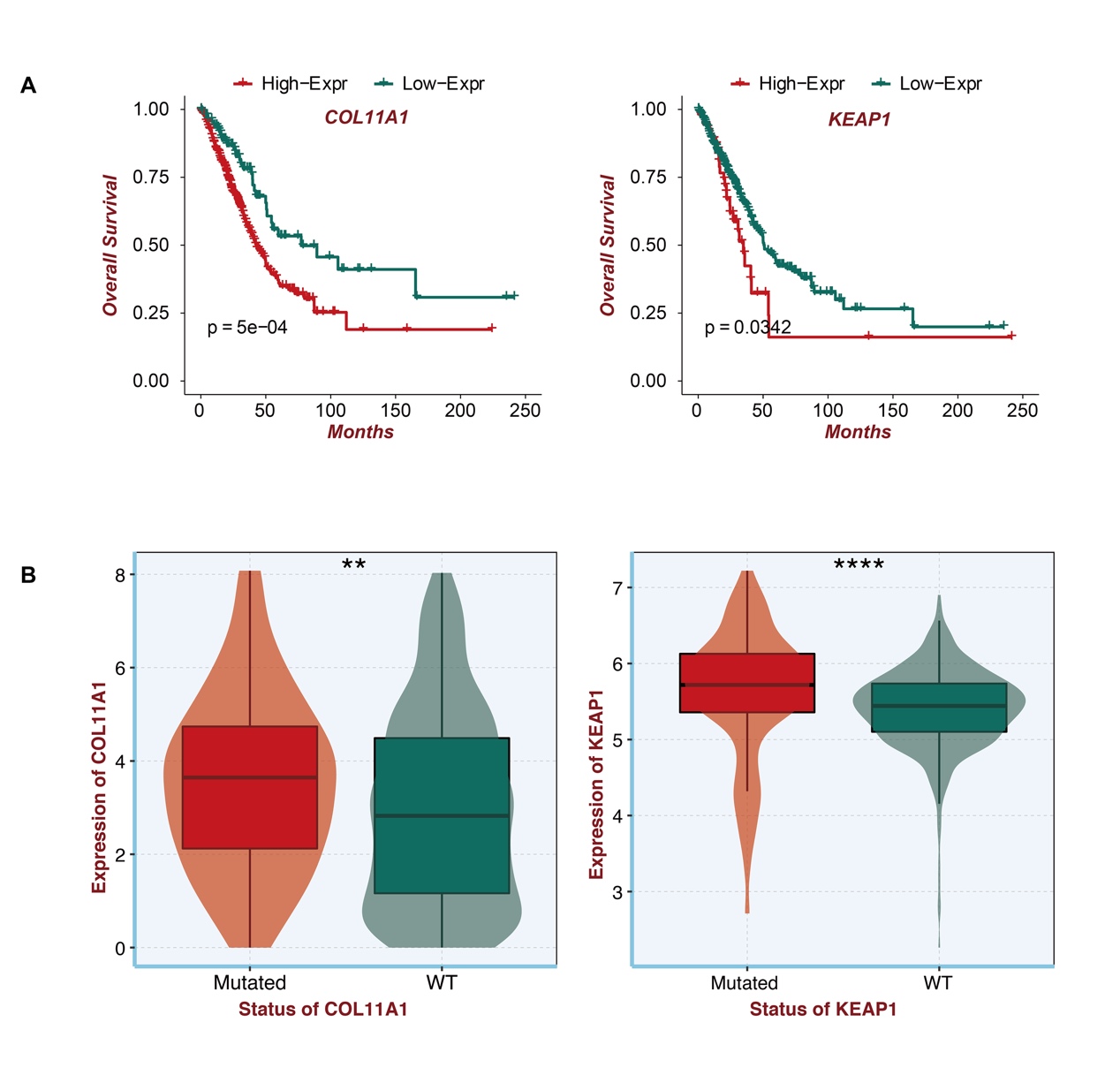


Figure S6. A. Kaplan-Meier survival curves of COL11A1 and KEAP1 regarding OS. B. The association between COL11A1 and KEAP1 expression and COL11A1 and KEAP1 mutation.


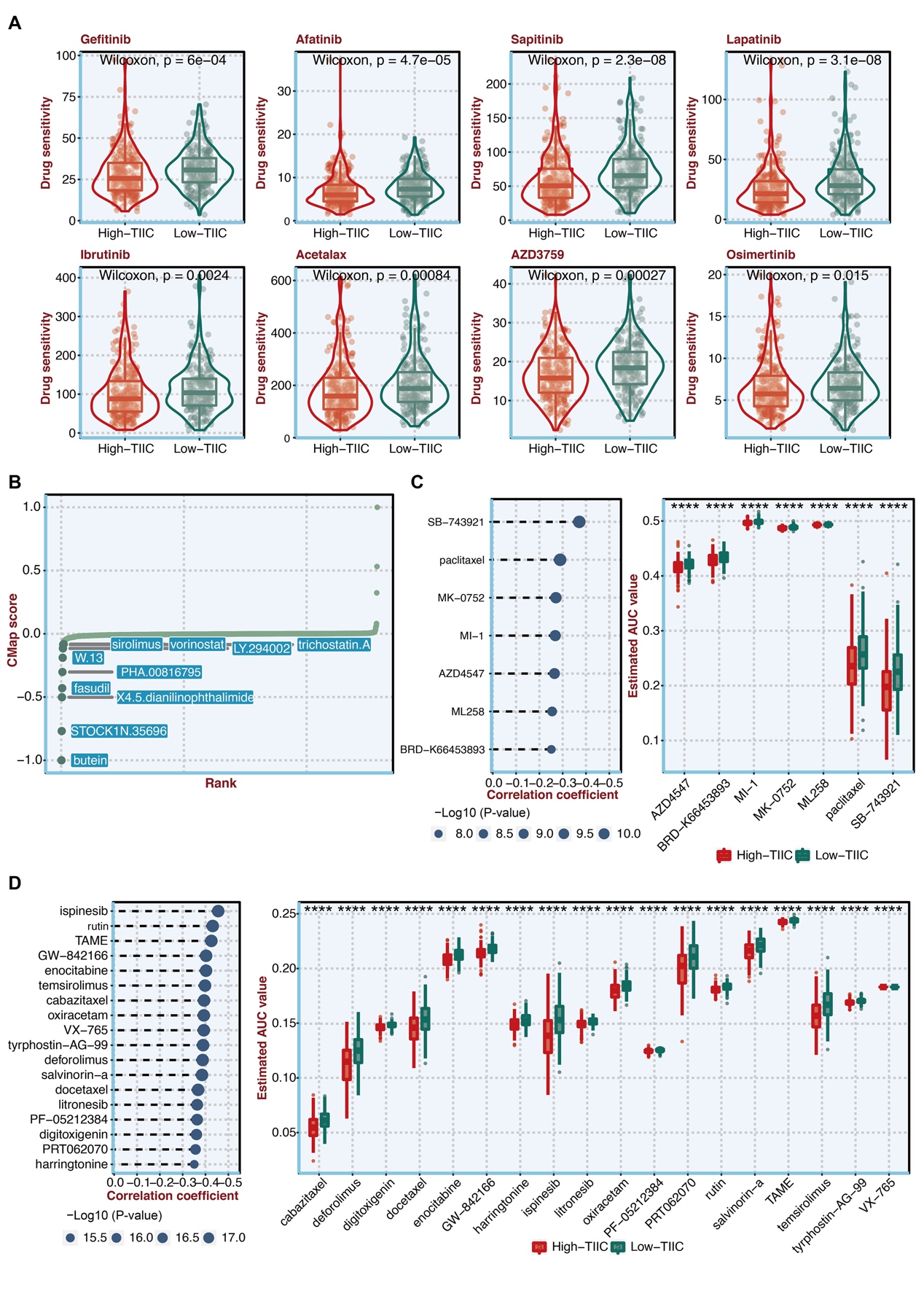


Figure S7. The drug responses between two TIIC signature score groups. A. GDSC-based drug prediction. B. CMap-based drug prediction. C. CTRP-based drug prediction. D. PRISM-based drug prediction.
